# Supplementary material for: The Ameliorative Effect of COST on Diet-Induced Lipid Metabolism Disorders by Regulating Intestinal Microbiota
Source: Mar Drugs. 2022 Jul 7;20(7):444. doi: 10.3390/md20070444 (PMC9317995; doi:10.3390/md20070444)
Supplement: Supplementary file 1 [file marinedrugs-20-00444-s001.zip › marinedrugs-1782769-supplementary.pdf]

Table S1 : Effects of COST on body weight and various physiological and biochemical parameters in mice with disorders of lipid metabolism, correlated with Figure 1 (n = 6). Data are expressed as mean ± SEM and statistically analyzed via one-way ANOVA; *$p < 0.05$, **$p < 0.01$, ***$p < 0.001$; **** $p < 0.0001$.

	**CTRL**	**MOD**	**COST-H**	**COST-M**	**COST-L**
Body weight(g)	26.76±0.083***	33.62±0.48	32.28±0.56	33.10±0.60	33.64±0.89
Serum TC(mmol/L)	3.90±0.13****	5.66±0.20	4.35±0.17****	4.59±0.12**	4.98±0.26*
Serum TG(mmol/L)	0.33±0.04****	0.67±0.03	0.48±0.02**	0.47±0.03**	0.52±0.04*
Serum HDL-C(mmol/L)	7.84±0.31****	4.70±0.06	6.84±0.26****	6.42±0.25***	5.52±0.10
Serum LDL-C(mmol/L)	1.83±0.07****	3.60±0.21	2.45±0.15***	2.67±0.15**	3.34±0.26
Serum NEFA (mmol/L)	0.53±0.01****	1.04±0.02	0.56±0.02****	0.61±0.023****	0.64±0.026****
Serum GLU (mmol/L)	5.51±0.11****	12.23±0.34	9.18±0.47****	10.85±0.0.53	11.62±0.55

Table S2: Mice body weight, serum and liver biochemical markers, and liver and adipose tissue weight during FMT, correlated with Fiure 3, Figure 4 and Figure 5 (n = 6).Data are expressed as mean ± SEM and statistically analyzed via one-way ANOVA; *$p < 0.05$, **$p < 0.01$, ***$p < 0.001$; **** $p < 0.0001$.

	**STD**	**Control**	**Model**	**COST-H-FMT**	**COST-M-FMT**	**COST-L-FMT**
Body weight(g)	28.84±0.55***	29.85±0.52*	32.49±0.50	26.19±0.65***	27.38±0.71***	27.88±0.65***
LiverWeight(g)	0.9807±0.05**	1.0156±0.04*	1.1457±0.03	0.9214±0.02***	0.9391±0.03**	0.8758±0.03***
Brown adipose tissue (g)	0.132±0.01	0.125±0.01	0.098±0.00	0.118±0.00	0.109±0.01	0.108±0.01
Subcutaneous adipose	0.199±0.01***	0.400±0.04*	0.557±0.04	0.207±0.03***	0.326±0.04***	0.352±0.05**
Perirenal adipose	0.071±0.01***	0.210±0.03***	0.422±0.04	0.150±0.02***	0.175±0.03***	0.194±0.03***
Epididymal adipose	0.344±0.02***	0.789±0.10***	1.325±0.09	0.540±0.10***	0.601±0.07***	0.652±0.08***
Fat/body ratio (%)	2.58%***	5.08%***	7.41%	3.88%***	4.43%***	4.68%***
Serum TC(mmol/L)	7.84±0.28***	6.89±0.52***	10.30±0.42	7.34±0.19***	7.71±0.37***	7.96±0.42**
Serum TG(mmol/L)	0.95±0.03*	0.83±0.13**	1.45±0.16	0.50±0.16***	0.77±0.07**	1.04±0.08
Serum HDL-C(mmol/L)	3.76±0.01***	3.14±0.16	2.56±0.16	4.26±0.15***	4.22±0.21***	3.61±0.21***
Serum LDL-C(mmol/L)	0.28±0.04***	0.95±0.15***	2.40±0.38	0.90±0.19***	0.87±0.08***	1.22±0.15**
Serum ALT(mmol/L)	82.72±3.88***	45.38±5.85***	131.16±10.71	50.16±2.39***	50.16±5.85***	46.99±0.48***
Serum AST(mmol/L)	84.30±3.80**	79.42±2.16	106.40±1.52	67.73±2.46***	76.90±2.98	77.10±2.78
Serum Glucose(mmol/L)	8.06±0.37**	7.23±0.44***	11.02±0.42	6.35±0.47***	8.02±0.22***	7.99±0.36***
Serum NEFA (mmol/L)	0.94±0.06***	1.07±0.02*	1.25±0.04	0.75±0.04***	0.86±0.04***	0.99±0.03***
Serum TBA (mmol/L)	2.31±0.16***	4.37±0.16	5.28±0.46	3.04±0.18***	3.30±0.14***	4.16±0.25*
Liver TG level(mmol/L)	3.47±0.51***	5.80±1.10**	9.04±2.42	2.96±0.31***	4.99±0.96**	5.04±1.46**
Liver TC level(mmol/L)	1.47±0.39**	1.98±0.15*	2.69±0.40	1.68±0.411**	1.70±0.23*	2.16±0.28
Liver LDL- C level	0.38±0.076**	0.44±0.097	0.67±0.060	0.38±0.087*	0.39±0.092*	0.47±0.11
Liver TBA level	59.13±8.89***	86.13±11.77*	123.33±18.36	66.90±13.43***	78.74±19.06**	83.98±22.45**

Table S3 Animal Feed Information

Feed	Protein （%）	Carbohydrate （%）	FAT （%）	Fiber （%）	Calcium/Phosphorus （%）
Standard formula feed	20	0	4.3	4.8	1.19
D12327 High-fat and high-sugar feed	23	46.1	20.4	5.5	5

Table S4 Primer sequence

Gene	Forward primer	Reverse primer
SREBP	5'-CAGCTATTGGCCTTCCTCAG-3'	5'-GGTTACTGGCGGTCACTGTC-3'
FAS	5'-ATGCACACTCTGCGATGAAG-3'	5'-TTCAGGGTCATCCTGTCTCC-3'
PPARγ	5'-AGACCACTCGCATTCCTTTG-3'	5'-CATTGGGTCAGCTCTTGTGA-3'
PPARα	5'-GCAGCTCGTACAGGTCATCA-3'	5'-ACTGCCGTTGTCTGTCACTG-3'
CYP7A1	5'-GGGATTGCTGTGGTAGTGAGC-3'	5'-GGTATGGAATCAACCCGTTGTC-3'
FXR	5'-GCTTGATGTGCTACAAAAGCTG-3'	5'-GTGGTGATGGTTGAATGTCC-3'
Occludin	5'-ATGTCCGGCCGATGCTCTC-3'	5'-TTTGGCTGCTCTTGGGTCTGTAT-3'
Claudin	5'-TCTACGAGGGACTGTGGATG-3'	5'-TCAGATTCAGCTAGGAGTCG-3'
ZO1	5'-ACCCGAAACTGATGCTGTGGATAG-3'	5'-AAATGGCCGGGCAGAACTTGTGTA-3'
β-Actin	5'-CGTGAAAAGATGACCCAGA-3'	5'-GTCCATCACAATGCCTGT-3'

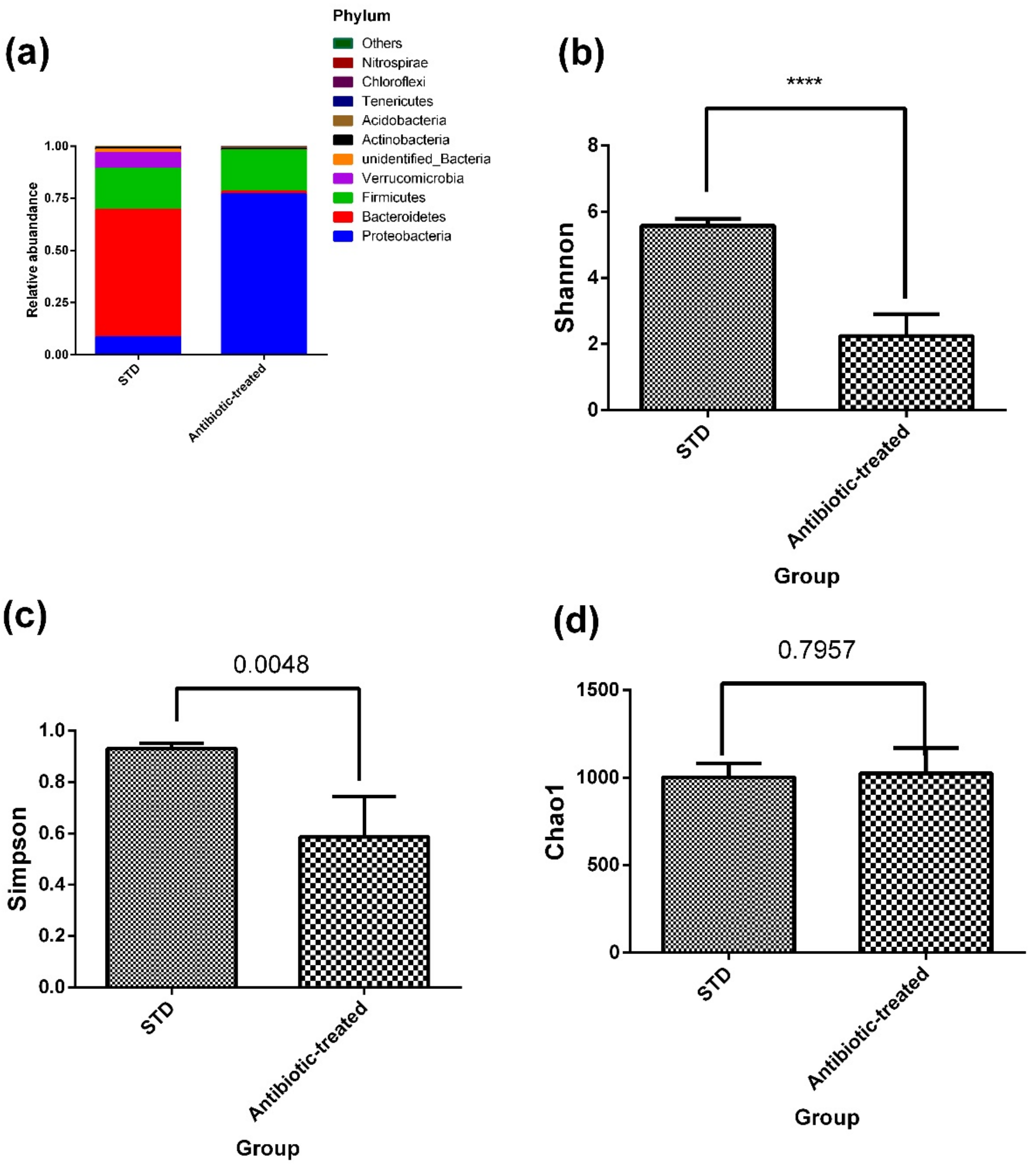

Figure S1: Species levels of phylum differences in mice following antibiotic intervention (a), and changes in Alpha index levels (**b-d**)(n = 6).
